# Patient and Hospital Factors Associated With Unexpected Newborn Complications Among Term Neonates in US Hospitals

**DOI:** 10.1001/jamanetworkopen.2019.19498

**Published:** 2020-02-05

**Authors:** Mark A. Clapp, Kaitlyn E. James, Sara V. Bates, Anjali J. Kaimal

**Affiliations:** Department of Obstetrics and Gynecology, Massachusetts General Hospital, Boston; Harvard Medical School, Harvard University, Boston, Massachusetts; Department of Obstetrics and Gynecology, Massachusetts General Hospital, Boston; Harvard Medical School, Harvard University, Boston, Massachusetts; Department of Pediatrics, Massachusetts General Hospital, Boston; Department of Obstetrics and Gynecology, Massachusetts General Hospital, Boston; Harvard Medical School, Harvard University, Boston, Massachusetts

## Abstract

**IMPORTANCE:**

Unexpected complications in term newborns have been recently adopted by the Joint Commission as a marker of obstetric care quality.

**OBJECTIVE:**

To understand the variation and patient and hospital factors associated with severe unexpected complications in term neonates among hospitals in the United States.

**DESIGN, SETTING, AND PARTICIPANTS:**

This cross-sectional study collected data from all births in US counties with 1 obstetric hospital using county-identified birth certificate data and American Hospital Association annual survey data from January 1, 2015, through December 31, 2017. All live-born, term, singleton infants weighing at least 2500 g were included. The data analysis was performed from December 1, 2018, through June 30, 2019.

**EXPOSURES:**

Severe unexpected newborn complication, defined as neonatal death, 5-minute Apgar score of 3 or less, seizure, use of assisted ventilation for at least 6 hours, or transfer to another facility.

**MAIN OUTCOMES AND MEASURES:**

Between-hospital variation and patient and hospital factors associated with unexpected newborn complications.

**RESULTS:**

A total of 1 754 852 births from 576 hospitals were included in the analysis. A wide range of hospital complication rates was found (range, 0.6–89.9 per 1000 births; median, 15.3 per 1000 births [interquartile range, 9.6–22.0 per 1000 births]). Hospitals with high newborn complication rates were more likely to care for younger, white, less educated, and publicly insured women with more medical comorbidities compared with hospitals with low complication rates. In the adjusted models, there was little effect of case mix to explain the observed between-county variation (11.3%; 95% CI, 10.0%−12.6%). Neonatal transfer was the primary factor associated with complication rates, especially among hospitals with the highest rates (66.0% of all complications). The risk for unexpected neonatal complication increased by more than 50% for those neonates born at hospitals without a neonatal intensive care unit compared with those with a neonatal intensive care unit (adjusted odds ratio, 1.55; 95% CI, 1.38–1.75).

**CONCLUSIONS AND RELEVANCE:**

In this study, severe unexpected complication rates among term newborns varied widely. When included in the metric numerator, neonatal transfer was the primary factor associated with complications, especially among hospitals with the highest rates. Transfers were more likely to be necessary when infants were born in hospitals with lower levels of neonatal care. Thus, if this metric is to be used in its current form, it would appear that accreditors, regulatory bodies, and payers should consider adjusting for or stratifying by a hospital’s level of neonatal care to avoid disincentivizing against appropriate transfers.

## Introduction

In obstetrics, 2 patients have outcomes resulting from the process of labor and delivery: mother and infant. To date, measures of obstetrical care quality have primarily focused on maternal outcomes. Examples of proposed and adopted hospital measures of obstetric care include rates of cesarean delivery, episiotomy, higher-order perineal laceration, trial of labor after cesarean delivery, and postpartum readmissions.^1–7^ Of these, the cesarean delivery rate has been studied extensively and widely adopted and endorsed as an important quality metric based on the idea that overuse of cesarean delivery unnecessarily exposes more women to the risks of surgical complications and affects their risks in subsequent pregnancies.

Little focus has been placed on the neonatal outcomes of labor and delivery. The most widely adopted obstetric quality metric aimed at reducing neonatal morbidity is avoidance of elective delivery before 39 weeks.^8,9^ In 2011, the California Maternal Quality Care Collaborative developed a novel neonatal metric to serve as a balancing measure to more maternal-focused metrics of intrapartum care.^10^ This metric, Unexpected Complications in Term Newborns, captures adverse neonatal conditions that may be associated with labor and delivery management. On January 1, 2019, the Joint Commission implemented this metric as part of their Perinatal Core Measures, and as such, hospitals will now be asked to report their rate of unexpected complications.^8^ This measure has been proposed to serve as a balancing measure to maternal metrics, such as the rate of nulliparous, term, singleton, vertex-presenting cesarean deliveries.^11–17^

The objective of this study was to examine the distribution of unexpected complication rates in term newborns across the United States, to determine whether significant variation exists between hospitals, and to examine potential sources of variation and risk factors for complications. We hypothesized that, compared with the maternal-focused cesarean delivery rate metric, complications would be overall rare events and therefore have narrow distribution and little variance between hospitals.

## Methods

### Data Sources

This cross-sectional study used data from the US Standard Certificate of Live Birth, obtained with permission from the National Center for Health Statistics.^18^ This complete data set was chosen because it contains detailed maternal, neonatal, and delivery information and is collected via a standardized form, allowing for uniform collection of information for the approximately 4 million births per year. The study was conducted using births from January 1, 2015, through December 31, 2017; by January 1, 2015, every state in the United States had adopted the 2003 revised form with the exception of Connecticut and New Jersey.^18^ At the time that this data analysis was conducted (December 1, 2018, through June 30, 2019), the 2017 natality data were the most recent available. The smallest unit of analysis that could be obtained was at the level of the county. The project was reviewed and exempted by the Partners Healthcare Human Research Committee. Informed consent was not required for this review of deidentified data. The methods and findings from this study are reported in accordance with the Strengthening the Reporting of Observational Studies in Epidemiology (STROBE) reporting guideline.

Hospital information was obtained from the 2015 American Hospital Association (AHA) annual survey.^19^ Specifically, the AHA survey data were used to quantify the number of hospitals in each county that reported beds designated for obstetric care and for higher levels of neonatal care.

### Defining the Outcome

Originally developed by the California Maternal Quality Care Collaborative (CMQCC), the Joint Commission implemented the perinatal quality metric Unexpected Complications in Term Newborns (PC-06) in 2019.^8,10^ The CMQCC/Joint Commission measure relies on administrative and electronic health record data, including codes from the *International Statistical Classification of Diseases and Related Health Problems, Tenth Revision*; we approximated unexpected severe complications among term newborns using information available on the birth certificate. We focused on severe complications because, per the CMQCC documentation, “severe unexpected newborn complications is where most attention should be focused,” and “severe unexpected newborn complications can be used as a balancing measure for QI [quality improvement] efforts to reduce primary or NTSV [nulliparous, term, singleton, vertex-presenting] cesarean birth rates.”^20(p2)^ In our analysis, the denominator was similar to the Joint Commission measure and defined as infants who were live-born (5-minute Apgar score >0), term (≥37 weeks’ gestation), singleton gestations, and nonanomalous, with a birth weight of at least 2500 g. Births listed as or intended as extramural deliveries were also excluded. To calculate the numerator, diagnosis codes are not listed on the birth certificate; however, the 2003 version of the birth certificate contains information on the occurrence of newborn complications. We considered assisted ventilation of at least 6 hours and seizure or serious neurological dysfunction to be severe and unlikely to represent false-positive complications. We also considered a 5-minute Apgar score of 3 or less as a severe complication, in accordance with other studies that assessed significant neonatal complications.^21–24^ Last, neonatal death and transfer to another facility were considered severe neonatal complications in line with the Joint Commission metric. A summary of the Joint Commission metric specifications and the data elements used from the birth certificate for this analysis are described in detail in the eMethods and eTable 1 in the Supplement.

### Hospital-Level Complication Rates

Unexpected complications in term newborns were calculated at the county level, the smallest unit of analysis available in the birth certificate data set. However, to understand hospital-level variation, counties with more than 1 hospital with obstetric beds were excluded, because we could not assign deliveries to specific hospitals within an individual county. Therefore, deliveries within the remaining counties were assumed to have occurred at the 1 hospital with obstetric beds. In accordance with the Joint Commission reporting guidelines for this metric, the rate was only calculated for hospitals with at least 300 deliveries per year for each year in the study period. The rate was calculated across the 3-year period to increase the denominator and more accurately reflect the true practice of the hospital. The rate was reported as number of newborns with complications per 1000 eligible births.

### Statistical Analysis

For comparison, hospitals were grouped into deciles by their complication rate. The maternal, newborn, delivery, and hospital characteristics were compared between hospitals with the lowest (first decile), middle (second through ninth deciles), and highest (tenth decile) complication rates. The following maternal characteristics were compared: age (categorized into 5-year increments), race, ethnicity, birth place, educational level, insurance payer, comorbidities (tobacco use, pregestational diabetes, gestational diabetes, chronic hypertension, and pregnancy-related hypertension), and parity. Race and ethnicity were examined because they have known associations with obstetric outcomes; these variables were categorized by the National Center for Health Statistics in the raw data. The following newborn and delivery characteristics were compared: gestational age (in weeks), infant birth weight (in grams), delivery mode, induction vs spontaneous labor, and maternal transfer. The following hospital characteristics were compared: percentage of Medicaid-covered births, mean annual delivery volume, percentage of county population living in rural areas (based on 2010 census data), and level of neonatal care (low vs high).^25,26^ Hospitals with neonatal intensive care unit (NICU) beds were considered to have a high level of neonatal care, and those without NICU beds were considered to have a low level of neonatal care. The NICU bed data were obtained from the 2015 AHA hospital survey; NICU and intermediate neonatal care beds were considered NICU beds for this analysis because term infants could be admitted to either at this gestational age for higher-level neonatal care. Last, the overall complication rate and the individual components of the complication were compared between the hospitals. We used χ^2^ tests, 2-sided *t* tests, and Wilcoxon rank sum tests for comparisons, when appropriate. All analyses were conducted in Stata/SE, version 14.1 (StataCorp LLC). Two-sided *P* < .05 was considered statistically significant.

### Individual and Hospital Risk Factors for Complications

Currently, the Joint Commission does not recommend additional risk adjustment for this metric. However, to determine whether specific maternal, delivery, or hospital factors were associated with a neonate’s risk for complication, we constructed a mixed-effects model using patient-level data. The model accounted for the random effect of the hospital and fixed effect of year, in addition to the maternal factors, neonatal factors, and hospital factors described earlier. These factors were selected a priori. Missing data were rare and appeared to be present at random; thus, complete case analyses were performed.

### Hospital-Level Variation

Hierarchical mixed-effects models using patient-level data and accounting for the random effect of the hospital were also used to estimate the amount of variation that could be attributed to systematic differences between the hospitals (eg, between-hospital variation). First, a model that only contained the fixed effect of year in addition to the random effect of the hospital was used. Then, the maternal factors and neonatal factors were added as a means of adjusting for the hospital’s case mix. Last, observed hospital variables were added to determine what proportion of the between-hospital variation remained. Intraclass coefficients from these logistic regression models were calculated based on previously described methods.^27^

### Neonatal Transfer and Level of Neonatal Care

The current metric considers transfer to another facility as a severe complication. We hypothesized that facilities with higher levels of neonatal care would have lower rates of transfer, because they have fewer indications for transfer. To understand this association, the distributions of hospital complication rates were plotted including and excluding neonatal transfers from the metric numerator. Furthermore, complication rates including and excluding neonatal transfer were compared between hospitals by neonatal level of care among common maternal comorbidities (pregestational diabetes, gestational diabetes, chronic hypertension, and pregnancy-induced hypertension). The amount of observed between-hospital variation explained by a hospital’s level of neonatal care was further examined by comparing the hierarchical mixed-effects model results with and without the NICU bed variable. At the individual level, we compared the association of neonatal level of care with risk of neonatal complication when transfers were included and excluded.

### Sensitivity Analyses

Sensitivity analyses were performed to evaluate the robustness and generalizability of the primary findings. To evaluate whether state-level policy or factors may be influencing care practices or the outcome, we controlled for the fixed effect of state in the hierarchical models. For the next sensitivity analysis, we attempted to exclude newborns born to mothers with substance use disorder, in which a newborn complication would not be unexpected, by restricting the analysis to nonusers of tobacco. Afterward, we excluded newborns born to mothers who were transferred after delivery; this approach removed newborns who may have been transferred to be in close proximity to their mothers rather than for the need for higher-acuity neonatal care. Last, to demonstrate the generalizability of the findings to counties with more than 1 obstetric hospital, all analyses were conducted at the county level in counties with more than 1 obstetric hospital. All sensitivity analyses and their rationale are described in detail in the eMethods in the Supplement.

## Results

From 2015 to 2017, 11 397 964 births occurred in the 48 states and District of Columbia that used the 2003 version of the US Standard Certificate of Live Birth. Of these, 9 618 598 neonates (84.4%) were term, singleton, nonanomalous, and live-born, with birth weights of at least 2500 g. Births of these infants were reported from 2841 counties. However, only 1063 counties had more than 300 hospital-based births per year (9 444 295 deliveries), of which 966 counties were reported to have at least 1 hospital with designated obstetric beds in the AHA hospital survey data (8 907 747 deliveries). Of the 97 counties with missing AHA hospital survey data, only 4 counties (4.1%) with a total of 10 143 deliveries had more than 300 deliveries per year. The final study sample consisted of 576 counties from 48 states that were identified as having 1 hospital providing obstetric care and reporting at least 300 deliveries per year (1 754 852 deliveries), enabling us to approximate a hospital-level analysis. The 393 counties with more than 1 obstetric hospital were compared in the sensitivity analysis (7 153 097 deliveries). The hospital complication rates ranged from 0.6 to 89.9 per 1000 newborns (median, 15.3 [interquartile range {IQR}, 9.6–22.0] per 1000 newborns).

Hospitals that were in the lowest decile had less than 5.4 complications per 1000 newborns, and hospitals in the highest had more than 30.1 complications per 1000 newborns. Table 1 compares the maternal, delivery, and hospital characteristics that vary among those with low, middle, and high complication rates.

Table 2 lists the number and percentage of births with unexpected complication rates and the rates of the individual conditions constituting the metric. The complication rates were 3.6 and 37.9 per 1000 births in hospitals with the lowest and highest complication rates, respectively. The most common component of the composite was neonatal transfer, which occurred in 512 of 1244 complications (41.2%) in hospitals with low rates and 3007 of 4556 (66.0%) in hospitals with high rates.

**Figure 1.**
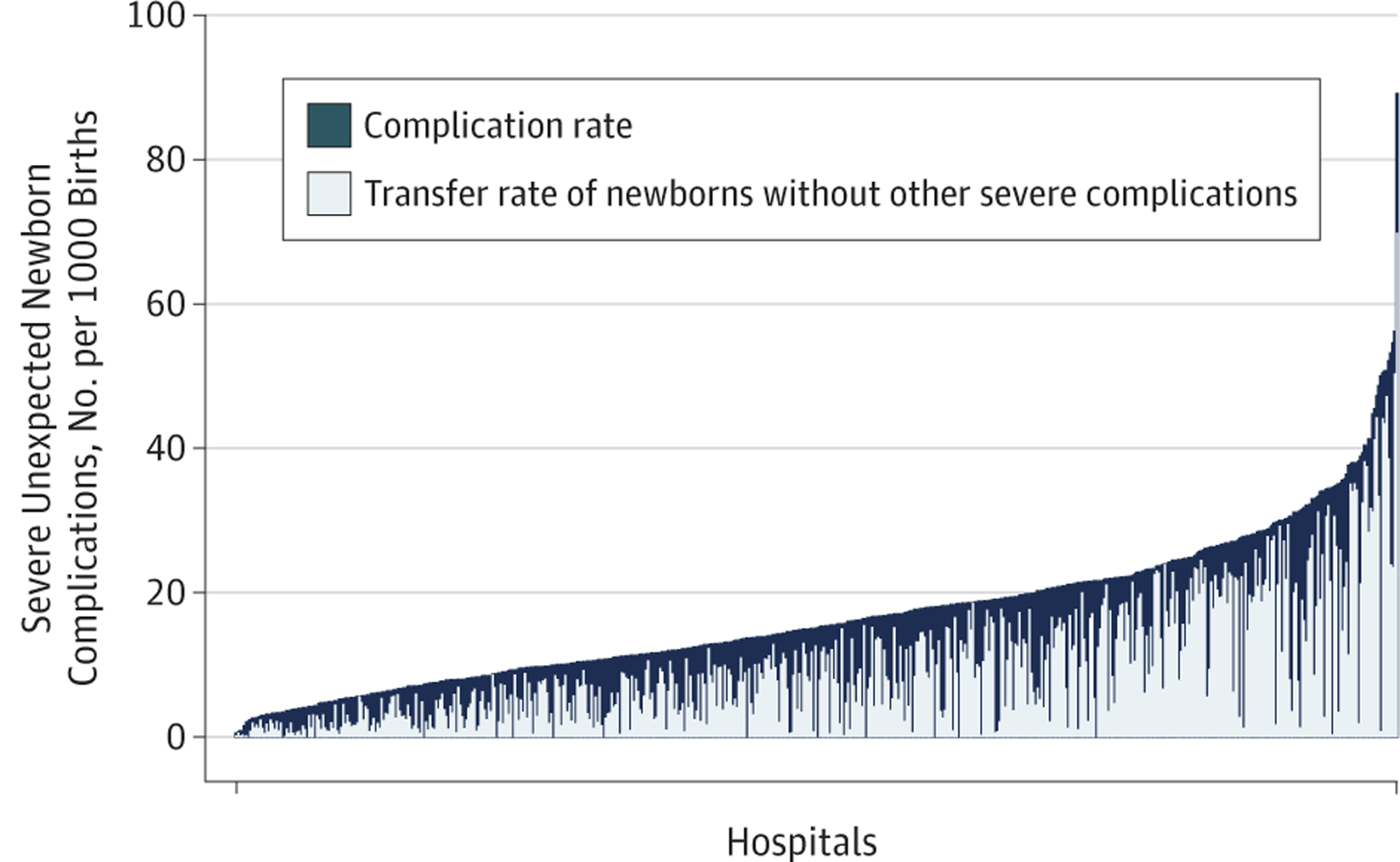
Severe Unexpected Newborn Complication and Neonatal Transfer Rates by Hospital The dark blue bars represent a hospital’s severe unexpected complication rate, which considers neonatal transfer to be a severe complication. The overlying light blue bars show the relative contribution of neonatal transfer (ie, the number of newborns transferred without another severe complication) to the overall complication rate.

Hospital complication rates were plotted, showing the relative contribution of neonatal transfers to the metric (Figure 1). Transfers constituted the most cases of unexpected complications, especially among hospitals with high complication rates. When transfers were excluded from the metric numerator, the distribution of complication rates shifted leftward (median rate decreased from 15.3 to 5.1 per 1000 births) and the IQR decreased to 3.1 to 9.0 per 1000 births.

Notable between-hospital variation occurred in unexpected complication rates before any adjustments (intraclass coefficient, 11.7%; 95% CI, 10.4%−13.0%). Little change was found after adjustments for case mix; 11.3% (95% CI, 10.0%−12.6%) of the hospital-level variation was attributed to systemic differences between hospitals. When observed county and hospital factors were added, the variation was slightly reduced to 8.8% (95% CI, 7.8%−9.9%).

In the patient-level analysis, maternal comorbidities were most consistently associated with an increased risk of neonatal complication (Table 3). The adjusted odds ratio (aOR) for complications among women with pregestational diabetes was 2.97 (95% CI, 2.73–3.24); among those with gestational diabetes, 1.36 (95% CI,1.29–1.43). This was similar for women with chronic hypertension (aOR, 1.47; 95% CI, 1.35–1.59) and pregnancy-induced hypertension (aOR, 1.51; 95% CI, 1.44–1.59). The aOR for the hospital factors in the patient-level analyses were 1.00 (95% CI, 1.00–1.00) for delivery volume and 1.00 (95% CI, 1.00–1.01) for percentage of rural population and not significant for Medicaid-covered deliveries (aOR, 0.91; 95% CI, 0.68–1.21). However, for level of neonatal care, the aOR for unexpected complication among births in hospitals without a NICU compared with those in a hospital with a NICU was 1.55 (95% CI, 1.38–1.75).

**Figure 2.**
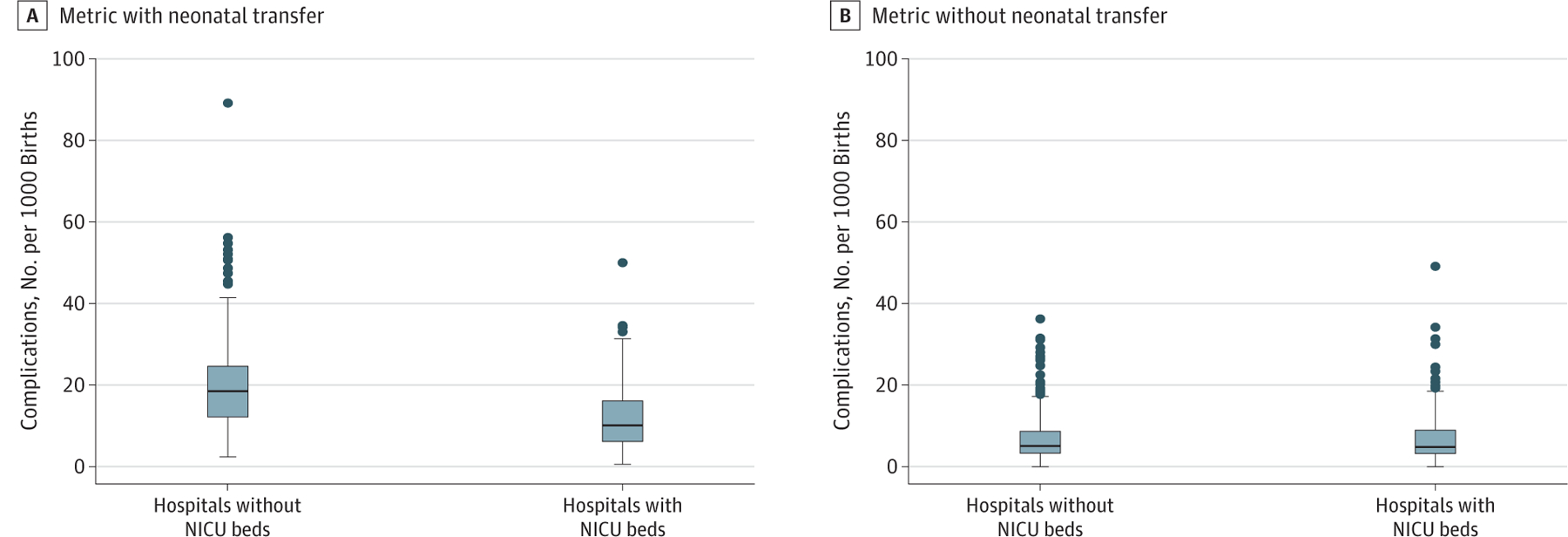
Distribution of Hospital Complication Rates With and Without Neonatal Transfer Center line indicates median; lower and upper borders of box, interquartile range; whiskers, upper (75th percentile + 1.5) and lower (25th percentile − 1.5) adjacent values; and circles, outliers. NICU indicates neonatal intensive care unit.

When stratified by level of neonatal care, the neonatal complication rate was 18.6 per 1000 births in hospitals without a NICU and 10.1 per 1000 births in hospitals with a NICU (*P* < .001). When transfers were excluded from the metric numerator, there was no difference between the 2 groups, with complication rates of 5.1 per 1000 births and 4.8 per 1000 births in counties without and with NICUs, respectively (*P* = .61) (Figure 2).

This association was further demonstrated among newborns born to mothers with medical comorbidities (hypertension and diabetes) (eFigure 1 in the Supplement). Complication rates among neonates born to women with these conditions were higher in hospitals without NICUs. However, when transfers were excluded from the metric numerator, complication rates were more similar or lower for these newborns in hospitals without compared with those with NICU beds (eFigure 1 in the Supplement). In the patient-level analysis, no association with neonatal level of care and the risk for a complication when transfer was excluded from the metric numerator was found for births in hospitals without a NICU compared with those with a NICU (aOR, 1.05; 95% CI, 0.89–1.24) (eTable 2 in the Supplement). Similar findings were demonstrated in the sensitivity analyses that included a state-level fixed effect, excluded neonates born to tobacco users, excluded neonates born to mothers who were transferred, and in counties with more than 1 obstetric hospital (eTables 3-9 and eFigures 2-4 in the Supplement).

## Discussion

Overall, unexpected complications in term newborns are uncommon; however, this study showed a wide range of hospital complication rates with measurable between-hospital variation. In the adjusted models, there was little effect of case mix to explain the observed between-hospital variation. Notably, neonatal transfer, which can impose a significant burden on families, was the most common complication. Transfer can occur for a variety of reasons, including for some of the other adverse outcomes in this composite. However, hospitals with higher levels of neonatal care are less likely to need to transfer infants because they are more likely to have the resources to care for more newborns with complications. In the patient-level analysis, a patient’s risk for an unexpected complication was increased by 50% when born in a hospital without a NICU; however, there was no increased risk when transfer was not considered a complication.

The Unexpected Complication in Term Newborns is the first metric adopted by the Joint Commission that measures neonatal outcomes after delivery. Developed by the CMQCC, endorsed by the National Quality Forum and now the Joint Commission, unexpected newborn complications have been largely understudied. Previously, the most comprehensive analysis was reported by Sebastião et al^28^ in 2017, who examined this metric using linked birth certificate and discharge records from 2004 to 2013 in Florida. In their study of 124 hospitals, they reported complication rates of 6.7 to 98.6 per 1000 births and noted transfers ranged from 0 to 67 per 1000 in hospitals with a low level of neonatal care compared with a range of 0 to 3 per 1000 in hospitals with a high level of neonatal care.^28^ We replicated many of the findings of Sebastião et al^28^ in our contemporary cohort of more than 1.7 million newborns from more than 500 hospitals across the United States and in more than 7 million newborns in the county-level sensitivity analysis. Notably, we focused only on severe complications, which are more likely to have serious implications for neonates and their families and as per the recommendation of the CMQCC when considering a balancing quality metric to maternal outcomes.

Given the recent adoption of this metric by the Joint Commission, these findings raise concern for smaller, rural, and community hospitals, which may have appropriately low levels of neonatal care for otherwise healthy women and neonates. Although a neonatal transfer often represents a significant burden on families in the immediate newborn period, it may also represent appropriate care to ensure the neonate receives necessary treatment. Furthermore, women with expected indications for a higher level of neonatal care, even among this low-risk population, may not have the means or ability to easily travel to another hospital with a higher level of neonatal care before delivery, thus necessitating postnatal transfer. Notably, the metric does not consider NICU admission a severe unexpected newborn outcome, effectively not penalizing more resourced referral hospitals that do not have a need to transfer infants to admit them to the NICU. Ideally, knowing the indication for transfer could better characterize the severity of the newborn outcome to determine whether it is equitable to the other components of the severe unexpected complication metric. These findings suggest that the association between transfer and neonatal care level should be considered if hospital benchmarking and public reporting is planned to avoid disproportionately penalizing those facilities with lower levels of neonatal care.

### Limitations

This study is, to our knowledge, the first nationwide analysis of severe unexpected complications in term newborns. The metric is generated from discharge diagnosis codes and clinical data (eg, birth weight, gestational age) and is difficult to study using commonly available national discharge databases because these data sets do not link maternal and infant records and lack clinical data.^29^ We approximated this metric using birth certificate data, which contains highly granular maternal and neonatal information. Although many data elements on the birth certificate have been proven to be accurate, data are lacking on the validity of newborn complications; thus, our results may be biased if complications are misreported. We were unable to include severe infection-related complications in the metric. However, we hypothesized that the inclusion of Apgar data and ventilation time likely captures severe complications not otherwise specified in our derived composite complication and approximates the Joint Commission metric. The Joint Commission also identifies a larger set of conditions to be moderate unexpected complications; we were unable to approximate moderate complications given the limited newborn data reported on the birth certificate.

The smallest unit of analysis available was the county for the birth certificate data; to perform a hospital-based analysis, we assumed that analyzing counties that had only 1 hospital reporting obstetric beds in the AHA survey was representative of a hospital analysis. This approach may limit the generalizability of our findings, because the subset of counties with only 1 obstetric hospital may have different patient-, hospital-, and county-level characteristics, which could potentially influence the results. In an attempt to address this issue, we replicated similar variation and associations with neonatal transfer in counties with more than 1 obstetric hospital. We may have misclassified the number of hospitals with obstetric and neonatal care services if hospitals did not respond to the survey; however, the AHA reports the inclusion of nearly 6400 hospitals and response rates of greater than 75%.^19^ Ideally, a nationwide hospital-level analysis should be performed, although no publicly available data source currently exists for this type of analysis, to our knowledge.

## Conclusions

There is wide variation in severe unexpected complication rates among term newborns. However, when using the current definition, neonatal transfer is the primary factor associated with complications, especially among hospitals with the highest rates. Transfers occur more commonly when infants are born in hospitals without a NICU. As a quality metric, hospitals with lower levels of neonatal care may be disproportionately penalized, which may in turn further limit women’s access to maternity services in community-based or rural hospitals or prompt hospitals to consider increasing their level of neonatal care or NICU capacity to avoid transfers. Thus, if this metric is to be used for performance evaluation or benchmarking, it appears that accreditors, regulatory bodies, and payers should consider a hospital’s level of neonatal care, either by risk adjustment or stratification, to avoid disincentivizing appropriate transfers.

## Supplementary Material

supplement **eMethods.** Severe Unexpected Newborn Complication Metric Approximation and Sensitivity Analyses
**eTable 1.** Joint Commission Measure Approximation of Severe Unexpected Newborn Complication Using Birth Certificate Data Elements
**eTable 2.** Adjusted Odds of Severe Unexpected Newborn Complication, Excluding Neonatal Transfer in the Metric Numerator, in the Patient-Level Analysis
**eTable 3.** Adjusted Odds of Severe Unexpected Newborn Complication in Patient-Level Analysis in the Model Including a State-Level Fixed Effect
**eTable 4.** Adjusted Odds of Severe Unexpected Newborn Complication in Patient-Level Analysis Among Nonusers of Tobacco
**eTable 5.** Adjusted Odds of Severe Unexpected Newborn Complication in Patient-Level Analysis Excluding Maternal Transfers
**eTable 6.** Comparison on Maternal, Delivery, and Hospitals Characteristics Between Counties With 1 vs >1 Obstetric Hospital
**eTable 7.** Comparison of Neonatal Complications Between Counties With 1 vs >1 Obstetric Hospital
**eTable 8.** Between-County Variation Estimations in Counties With >1 Obstetric Hospital
**eTable 9.** Adjusted Odds of Severe Unexpected Newborn Complication in the Patient-Level Analysis in Counties With >1 Obstetric Hospital
**eFigure 1.** Comparison of Complication Rates (Including and Excluding Neonatal Transfers From the Metric Numerator) by Comorbidity and by Level of Neonatal Care
**eFigure 2.** Distribution of Hospital Rates of Severe Unexpected Newborn Complications Among All Women and Among Nonusers of Tobacco
**eFigure 3.** Distribution of Hospital Rates of Severe Unexpected Newborn Complications Including and Excluding Maternal Transfers From the Metric Denominator
**eFigure 4.** Comparison of Distributions of Complication Rates Including and Excluding Transfer From the Metric Numerator Among Counties With >1 Obstetric Hospital
eReferences.

## Figures and Tables

**Table 1.** Comparison of Maternal, Delivery, and Hospital Characteristics Among Hospitals by Unexpected Newborn Complication Rates^a^

Characteristic	Hospital Neonatal Complication Rate^b^		
	Low	Middle	High
No. of deliveries	349 594	1 285 203	120 055
No. of hospitals	58	461	57
Maternal			
Age, y			
<18	4514 (1.3)	22 571 (1.8)	2404 (2.0)
18–24	92 400 (26.4)	405 791 (31.6)	41 002 (34.2)
25–29	106 296 (30.4)	407 651 (31.7)	37 973 (31.6)
30–34	93 402 (26.7)	302 602 (23.5)	26 212 (21.8)
35–39	43 837 (12.5)	123 316 (9.6)	10 506 (8.8)
≥40	9145 (2.6)	23 272 (1.8)	1958 (1.6)
Race			
White	259 877 (74.3)	1 065 059 (82.9)	100 431 (83.7)
Black	70 658 (20.2)	162 073 (12.6)	13 665 (11.4)
Native American/Alaskan	2894 (0.8)	19 303 (1.5)	3117 (2.6)
Asian or Pacific Islander	16 165 (4.6)	38 768 (3.0)	2842 (2.4)
Ethnicity			
Hispanic	49 637 (14.2)	179 469 (14.0)	17 156 (14.3)
Missing	1630 (0.5)	4359 (0.3)	496 (0.4)
Educational level			
Less than high school	41 231 (11.8)	175 805 (13.7)	19 473 (16.2)
High school	94 946 (27.2)	371 355 (28.9)	38 522 (32.1)
Any postsecondary	210 423 (60.2)	730 566 (56.8)	61 611 (51.3)
Missing	2994 (0.9)	7477 (0.6)	449 (0.4)
Payer at time of delivery			
Medicaid	144 797 (41.4)	584 349 (45.5)	65 235 (54.3)
Private	176 199 (50.4)	589 564 (45.9)	46 948 (39.1)
Self-pay	11 585 (3.3)	45 317 (3.5)	3564 (3.0)
Other	14 410 (4.1)	58 981 (4.6)	3779 (3.1)
Missing	2603 (0.7)	6992 (0.5)	529 (0.4)
Comorbidities			
Diabetes			
Pregestational	2717 (0.8)	8947 (0.7)	816 (0.7)
Gestational	17 049 (4.9)	70 211 (5.5)	7385 (6.2)
Hypertension			
Chronic	5093 (1.5)	20 391 (1.6)	2155 (1.8)
Pregnancy-induced	15 184 (4.3)	69 588 (5.4)	7191 (6.0)
Tobacco use	25 171 (7.2)	159 370 (12.4)	18 528 (15.4)
Missing	3776 (1.1)	7854 (0.6)	636 (0.5)
Parity			
Nulliparous	115 192 (33.0)	397 612 (30.9)	35 976 (30.0)
Multiparous	232 783 (66.6)	883 289 (68.7)	83 280 (69.4)
Missing	1619 (0.5)	4302 (0.3)	799 (0.7)
Delivery			
Gestational age, mean (SD), wk	39.4 (1.5)	39.3 (1.5)	39.3 (1.5)
Delivery mode			
Vaginal	245 420 (70.2)	925 444 (72.0)	83 982 (70.0)
Cesarean	103 696 (29.7)	359 431 (28.0)	36 055 (30.0)
Missing	478 (0.1)	328 (<0.1)	18 (<0.1)
Induction of labor	91 743 (26.2)	388 634 (30.2)	39 342 (32.8)
Missing	122 (<0.1)	470 (<.01)	87 (0.1)
Infant birth weight, mean (SD), g	3410 (434)	3414 (436)	3405 (436)
Missing	15 (<0.1)	229 (<0.1)	12 (<0.1)
Maternal transfer	347 (0.1)	2152 (0.2)	602 (0.5)
Missing	42 (<0.1)	569 (<0.1)	73 (0.1)
Hospital			
Annual hospital delivery volume, median (IQR)	1623 (872–2771)	670 (457–1044)	458 (353–642)
Births covered by Medicaid, median (IQR), %	45.1 (31.7–53.0)	48.8 (36.4–60.2)	53.3 (41.6–66.9)
County population in rural area, median (IQR), %	23.9 (7.6–33.7)	38.4 (24.9–54.6)	46.0 (34.7–55.8)
Hospitals with NICU beds, No. (%)	45 (77.6)	160 (34.7)	<10 (<17.5)

Abbreviations: IQR, interquartile range; NICU, neonatal intensive care unit.

a Low rate indicates first decile; middle rate, second to ninth deciles; and high rate, tenth decile. Unless otherwise indicated, data are expressed as number (percentage) of deliveries. Percentages have been rounded and may not total 100.

b Per data use reporting guidelines, cell sizes less than 10 were suppressed.

**Table 2.** Comparison of Severe Unexpected Newborn Complications Among Hospitals With Low, Middle, and High Rates^a^

Severe Unexpected Newborn Complications	Hospital Neonatal Complication Rate, No. of Deliveries (Rate/1000 Births)		
	Low (n = 349 594)	Middle (n = 1 285 203)	High (n = 120 055)
All	1244 (3.6)	18 804 (14.6)	4556 (37.9)
Transfer	512 (1.4)	11 159 (8.7)	3007 (25.0)
Assisted ventilation ≥6 h	266 (0.8)	6333 (4.9)	1660 (13.8)
Seizure	29 (0.1)	542 (0.4)	56 (0.5)
Neonatal death	108 (0.3)	303 (0.2)	30 (0.2)
5-min Apgar score ≤3	477 (1.4)	3488 (2.7)	400 (3.3)
Missing	785 (2.2)	3051 (2.4)	124 (1.0)

a Low rate indicates first decile; middle rate, second to ninth deciles; and high rate, tenth decile.

**Table 3.** Adjusted Odds of Severe Unexpected Newborn Complications in the Patient-Level Analysis

Characteristic	aOR (95% CI)^a^	*P* Value
Maternal		
Maternal age, y		
<18	0.94 (0.85–1.04)	.28
18–24	1 [Reference]	NA
25–29	1.02 (0.98–1.05)	.30
30–34	1.03 (0.99–1.07)	.16
35–39	1.06 (1.01–1.11)	.03
≥40	1.21 (1.10–1.32)	<.001
Maternal race		
White	1 [Reference]	NA
Black	0.97 (0.93–1.02)	.21
Native American/Alaskan	0.93 (0.84–1.04)	.22
Asian or Pacific Islander	0.84 (0.77–0.91)	<.001
Maternal ethnicity		
Non-Hispanic	1 [Reference]	NA
Hispanic	0.76 (0.73–0.80)	<.001
Maternal educational level		
Less than high school	1.02 (0.97–1.06)	.45
High school	1 [Reference]	NA
Any postsecondary	0.93 (0.90–0.96)	<.001
Payer at time of delivery		
Private	1 [Reference]	NA
Medicaid	1.17 (1.13–1.21)	<.001
Self-pay	1.25 (1.16–1.36)	<.001
Other	1.11 (1.03–1.19)	.004
Maternal comorbidities		
Diabetes		
Pregestational	2.97 (2.73–3.24)	<.001
Gestational	1.36 (1.29–1.43)	<.001
Hypertension		
Chronic	1.47 (1.35–1.59)	<.001
Pregnancy-related	1.51 (1.44–1.59)	<.001
Tobacco use	1.31 (1.26–1.36)	<.001
Parity		
Nulliparous	1 [Reference]	NA
Multiparous	0.70 (0.68–0.72)	<.001
Delivery		
Gestational age at delivery, wk	0.94 (0.93–0.95)	<.001
Delivery mode		
Vaginal	1 [Reference]	NA
Cesarean	2.10 (2.05–2.16)	<.001
Induction of labor	0.90 (0.87–0.93)	<.001
Infant birth weight, g	1.00 (1.00–1.00)	.08
Hospital		
Delivery volume	1.00 (1.00–1.00)	.002
Medicaid-covered deliveries, %	0.91 (0.68–1.21)	.51
County population in rural areas, %	1.00 (1.00–1.01)	.02
Level of neonatal care		
High	1 [Reference]	NA
Low	1.55 (1.38–1.74)	<.001

Abbreviations: aOR, adjusted odds ratio; NA, not applicable.

a Accounts for the random effect of the hospital, the fixed effect of year, and for all covariates listed. The reference category for the maternal comorbidities includes women without those individual conditions.

## Key Points

### Question

Do severe unexpected newborn complication rates vary by hospital, and what is the source of variation?

### Findings

In this cross-sectional study of 1 754 852 births from 576 US hospitals, hospital rates of severe unexpected complications in term newborns ranged from 0.6 to 89.9 per 1000 births. Neonatal transfer was the most common complication and the primary factor associated with the complication rate for most hospitals, especially among those without higher levels of neonatal care.

### Meaning

As an obstetric care quality measure, the inclusion of neonatal transfer as a severe unexpected term newborn complication may be disproportionately associated with hospitals with lower levels of neonatal care.
