# Supplementary material for: Patient and Hospital Factors Associated With Unexpected Newborn Complications Among Term Neonates in US Hospitals
Source: JAMA Netw Open. Author manuscript; Available in PMC 2021 Dec 22. (PMC8693709; doi:10.1001/jamanetworkopen.2019.19498)
Supplement: supplement — eMethods. Severe Unexpected Newborn Complication Metric Approximation and Sensitivity Analyses eTable 1. Joint Commission Measure Approximation of Severe Unexpected Newborn Complication Using Birth Certificate Data Elements eTable 2. Adjusted Odds of Severe Unexpected Newborn Complication, Excluding Neonatal Transfer in the Metric Numerator, in the Patient-Level Analysis eTable 3. Adjusted Odds of Severe Unexpected Newborn Complication in Patient-Level Analysis in the Model Including a State-Level Fixed Effect eTable 4. Adjusted Odds of Severe Unexpected Newborn Complication in Patient-Level Analysis Among Nonusers of Tobacco eTable 5. Adjusted Odds of Severe Unexpected Newborn Complication in Patient-Level Analysis Excluding Maternal Transfers eTable 6. Comparison on Maternal, Delivery, and Hospitals Characteristics Between Counties With 1 vs >1 Obstetric Hospital eTable 7. Comparison of Neonatal Complications Between Counties With 1 vs >1 Obstetric Hospital eTable 8. Between-County Variation Estimations in Counties With >1 Obstetric Hospital eTable 9. Adjusted Odds of Severe Unexpected Newborn Complication in the Patient-Level Analysis in Counties With >1 Obstetric Hospital eFigure 1. Comparison of Complication Rates (Including and Excluding Neonatal Transfers From the Metric Numerator) by Comorbidity and by Level of Neonatal Care eFigure 2. Distribution of Hospital Rates of Severe Unexpected Newborn Complications Among All Women and Among Nonusers of Tobacco eFigure 3. Distribution of Hospital Rates of Severe Unexpected Newborn Complications Including and Excluding Maternal Transfers From the Metric Denominator eFigure 4. Comparison of Distributions of Complication Rates Including and Excluding Transfer From the Metric Numerator Among Counties With >1 Obstetric Hospital eReferences. [file NIHMS1722836-supplement-supplement.docx]

Patient and Hospital Factors Associated With Unexpected Newborn Complications Among Term Neonates in US Hospitals

**Online Supplement**

Table of Contents

[eMethods: Severe Unexpected Newborn Complication Metric Approximation and Sensitivity Analyses 2](#_Toc25225194)

[eTable 1: Joint Commission measure approximation of severe unexpected newborn complication using birth certificate data elements 4](#_Toc25225195)

[eTable 2: Adjusted odds of severe unexpected newborn complication, excluding neonatal transfer in the metric numerator, in the patient-level analysis 5](#_Toc25225196)

[eFigure 1: Comparison of complication rates (including and excluding neonatal transfers from the metric numerator) by comorbidity and by level of neonatal care 6](#_Toc25225197)

[eTable 3: Adjusted odds of severe unexpected newborn complication in patient-level analysis in the model including a state-level fixed effect 7](#_Toc25225198)

[eTable 4: Adjusted odds of severe unexpected newborn complication in patient-level analysis among non-tobacco users 8](#_Toc25225199)

[eFigure 2: Distribution of hospital rates of severe unexpected newborn complications among all women and among non-tobacco users 9](#_Toc25225200)

[eTable 5: Adjusted odds of severe unexpected newborn complication in patient-level analysis excluding maternal transfers 10](#_Toc25225201)

[eFigure 3: Distribution of hospital rates of severe unexpected newborn complications including and excluding maternal transfers from the metric denominator 11](#_Toc25225202)

[eTable 6: Comparison on maternal, delivery, and hospitals characteristics between counties with one versus more than one obstetric hospital 12](#_Toc25225203)

[eTable 7: Comparison of neonatal complications between counties with one versus more than one obstetric hospital 14](#_Toc25225204)

[eFigure 4: Comparison of distributions of complication rates including and excluding transfer from the metric numerator among counties with more than one obstetric hospital 15](#_Toc25225205)

[eTable 8: Between-county variation estimations in counties with more than one obstetric hospital 16](#_Toc25225206)

[eTable 9: Adjusted odds of severe unexpected newborn in the patient-level analysis in counties with more than one obstetric hospital 17](#_Toc25225207)

# eMethods: Severe Unexpected Newborn Complication Metric Approximation and Sensitivity Analyses

Severe Unexpected Newborn Complication Metric Approximation

Originally developed by the California Maternal Quality Care Collaborative, The Joint Commission adopted the Perinatal Quality Metric Unexpected Complications in Term Newborns (PC-06) in 2018.^1,2^ The denominator for this metric is full term newborns without preexisting conditions, defined as singleton infants born at ≥37 weeks gestation with birth weights ≥2,500g without congenital malformation, exposure to maternal illicit drug use, or other pre-existing fetal conditions. The numerator for this metric is neonatal complication, stratified by severity (moderate or severe). Severe complications include death, transfer to another acute care facility, diagnosis codes for severe birth trauma, asphyxia, shock, respiratory complications, infection, and neurologic conditions. Moderate complications are mostly based on a combination of prolonged length of stay with less severe neonatal complication diagnosis codes. A full list of the diagnosis codes and conditions is found is available on the Joint Commission website.^1^

We approximated unexpected severe complications among term newborns using information available on the birth certificate. We focused on severe complications because, per the CMQCC documentation, “severe unexpected newborn complications is where most attention should be focused” and “severe unexpected newborn complications can be used as a balancing measure for QI efforts to reduce primary or NTSV cesarean birth rates.”^2^ In our analysis, the denominator was similar to the Joint Commission measure and defined as infants that were live born (5-minute Apgar >0), term (≥37 weeks gestation), singleton gestations, non-anomalous, and with birth weights ≥2,500 g. Births listed as or intended as extramural deliveries were also excluded. No information was available on maternal drug use. To calculate the numerator, diagnosis codes are not listed on the birth certificate; however, the 2003 version of the birth certificate does contain information on the occurrence of the following newborn complications: assisted ventilation required immediately following delivery, assisted ventilation required for more than 6 hours, neonatal intensive care unit (NICU) admission, use of surfactant replacement therapy, antibiotics received by the newborn for suspected neonatal sepsis, and seizure or serious neurologic dysfunction. Of these, we considered assisted ventilation >6 hours and seizure or serious neurologic dysfunction serious and unlikely to represent a false positive complication. In addition to these complications, we also considered 5-minute Apgar <3 as a severe complication, in accordance with other studies that studied significant neonatal complications.^3–6^ Lastly, neonatal death and transfer to another facility were considered serious neonatal complications in line with the Joint Commission metric. A summary of the Joint Commission metric specifications and the data elements used from the birth certificate for this analysis are found in eTable 1.

The metric is applied only to infants that were born at the facility and excludes infants transferred in for care. The delivery institution completes the birth certificate record, which

prevents outcomes the attribution of neonatal outcomes to accepting facilities in this data set.

Sensitivity Analyses

In the first sensitivity analysis, a fixed effect for state was added to the hierarchical models to adjust for any potential clustering effects among women or birth outcomes within a state.

Women with substance use disorder are an excluded population from the Joint Commission metric, as infants born to these mothers may experience withdrawal symptoms and require additional assistance after birth (i.e., a neonatal “complication” is not unexpected). Information on illicit substance use exposure, such as opioids, is not captured on the birth certificate. As we were unable to specifically exclude these infants, the rates generated in this analysis may be higher in hospitals caring for more women with substance use disorders. As a sensitivity analysis, we excluded women with tobacco use, which is captured on the birth certificate. Up to 95% of pregnant women receiving medication-assisted treatment for opioid use disorder report having concurrent tobacco use.^7,8^ Thus by excluding all tobacco users, we also likely exclude the vast majority of women with substance use disorders, who could be biasing the rate and variation estimates. The analyses described in the main text were performed in this population.

Furthermore, we were unable to distinguish the indications for neonatal transfer in this data set. A small percentage of women had the complication of “maternal transfer” (0.2%). In the primary analyses, all neonatal transfers were considered to be for neonatal indications (i.e., a neonate was not transferred to be with the mother after the mother was transferred to another facility). We compared hospital unexpected newborn complication rates including and excluding maternal transfers from the metric denominator to understand the degree to which neonatal transfers for non-neonatal indications could bias the results. The between-hospital variation and patient-level analyses were performed when maternal transfers were excluded.

The main analysis only included counties with one obstetric hospital as to study hospital-level variation. The maternal, delivery, and county characteristics of counties with only one obstetric hospital were compared to counties with more than one obstetric hospital. To demonstrate the generalizability of the findings to counties with more than one obstetric hospital, county-level variation in unexpected newborn complication rates including and excluding neonatal transfers from the metric numerator were compared. Between-county variation was calculated using the same mixed effects models as described in the primary analysis. Similarly, the patient-level analysis was performed to assess for the risk factors for unexpected neonatal complications in counties with more than one obstetric hospital.

References

1. The Joint Commission. Specifications Manual for Joint Commission National Quality Measures. Perinatal Care - PC-06 v2018B. https://manual.jointcommission.org/releases/TJC2018B/MIF0393.html. Accessed May 16, 2019.

2. California Maternal Quality Care Collaborative. Unexpected Complications in Term Newborns. https://www.cmqcc.org/focus-areas/quality-metrics/unexpected-complications-term-newborns. Accessed May 16, 2019.

3. Casey BM, McIntire DD, Leveno KJ. The continuing value of the Apgar score for the assessment of newborn infants. *N Engl J Med*. 2001;344(7):467-471. doi:10.1056/NEJM200102153440701

4. Iliodromiti S, Mackay DF, Smith GCS, Pell JP, Nelson SM. Apgar score and the risk of cause-specific infant mortality: a population-based cohort study. *Lancet Lond Engl*. 2014;384(9956):1749-1755. doi:10.1016/S0140-6736(14)61135-1

5. Tita ATN, Landon MB, Spong CY, et al. Timing of elective repeat cesarean delivery at term and neonatal outcomes. *N Engl J Med*. 2009;360(2):111-120. doi:10.1056/NEJMoa0803267

6. Grobman WA, Rice MM, Reddy UM, et al. Labor Induction versus Expectant Management in Low-Risk Nulliparous Women. *N Engl J Med*. 2018;379(6):513-523. doi:10.1056/NEJMoa1800566

7. Chisolm MS, Fitzsimons H, Leoutsakos J-MS, et al. A Comparison of Cigarette Smoking Profiles in Opioid-Dependent Pregnant Patients Receiving Methadone or Buprenorphine. *Nicotine Tob Res*. 2013;15(7):1297-1304. doi:10.1093/ntr/nts274

8. Akerman SC, Brunette MF, Green AI, Goodman DJ, Blunt HB, Heil SH. Treating Tobacco Use Disorder in Pregnant Women in Medication-Assisted Treatment for an Opioid Use Disorder: A Systematic Review. *J Subst Abuse Treat*. 2015;52:40-47. doi:10.1016/j.jsat.2014.12.002

# eTable 1: Joint Commission measure approximation of severe unexpected newborn complication using birth certificate data elements

|  | Joint Commission Measure | Birth Certificate Data Element |
| --- | --- | --- |
| Criteria for severe neonatal complication | Death | Neonatal demise |
|  | Transfer to another acute care facility | Newborn transfer |
|  | ICD-10 diagnosis codes for:  Severe birth trauma, hypoxia, shock, respiratory complications, infection, neurologic complications | 5-minute Apgar ≤ 3  Newborn complications:  assisted ventilation ≥ 6 hours, seizure / serious neurologic dysfunction |
|  | Length of stay > 4 days and ICD-10 code for sepsis |  |
| Denominator | Liveborn | 5-minute Apgar > 0 |
|  | Singleton | Singleton |
|  | Birth weight ≥ 2,500 g  No maternal illicit substance use | Birth weight ≥ 2,500 g  *Sensitivity analysis – excluding maternal tobacco use |

# eTable 2: Adjusted odds of severe unexpected newborn complication, excluding neonatal transfer in the metric numerator, in the patient-level analysis

|  | Characteristic | Fully Adjusted Odds Ratio (95% CI) | p-value |
| --- | --- | --- | --- |
| Maternal  Characteristics | Maternal age (years) |  |  |
|  | <18 | 0.97 (0.84-1.11) | 0.63 |
|  | 18-24 | *Reference* |  |
|  | 25-29 | 0.97 (0.92-1.02) | 0.18 |
|  | 30-34 | 0.96 (0.91-1.01) | 0.12 |
|  | 35-39 | 0.99 (0.93-1.07) | 0.87 |
|  | 40+ | 1.10 (0.97-1.25) | 0.12 |
|  | Maternal race |  |  |
|  | White | *Reference* |  |
|  | Black | 1.07 (1.01-1.13) | 0.03 |
|  | American Indian/Alaskan Native | 0.90 (0.78-1.05) | 0.19 |
|  | Asian or Pacific Islander | 0.91 (0.81-1.01) | 0.07 |
|  | Ethnicity |  |  |
|  | Hispanic | 0.81 (0.76-0.86) | 0.00 |
|  | Maternal education |  |  |
|  | Less than high school | 0.97 (0.91-1.03) | 0.36 |
|  | High school | *Reference* |  |
|  | Any post-secondary | 0.95 (0.91-1.00) | 0.04 |
|  | Payer at time of delivery |  |  |
|  | Private | *Reference* |  |
|  | Medicaid | 1.14 (1.09-1.20) | 0.00 |
|  | Self-pay | 1.14 (1.01-1.28) | 0.03 |
|  | Other | 1.09 (0.99-1.20) | 0.08 |
|  | Maternal comorbidities |  |  |
|  | Diabetes |  |  |
|  | Pre-gestational | 2.49 (2.21-2.80) | 0.00 |
|  | Gestational | 1.30 (1.21-1.39) | 0.00 |
|  | Hypertension |  |  |
|  | Chronic | 1.49 (1.34-1.66) | 0.00 |
|  | Pregnancy-related | 1.51 (1.41-1.61) | 0.00 |
|  | Tobacco use | 1.18 (1.12-1.25) | 0.00 |
|  | Parity |  |  |
|  | Nulliparous | *Reference* |  |
|  | Multiparous | 0.63 (0.61-0.66) | 0.00 |
| Delivery Characteristics | Gestational age at delivery (weeks) | 0.95 (0.94-0.96) | 0.00 |
|  | Delivery mode |  |  |
|  | Vaginal | *Reference* |  |
|  | Cesarean | 2.36 (2.27-2.45) | 0.00 |
|  | Induction of labor | 0.99 (0.95-1.03) | 0.51 |
|  | Birth weight (grams) | 1.00 (1.00-1.00) | 0.01 |
| Hospital Characteristics | County delivery volume in sample | 1.00 (1.00-1.00) | 0.89 |
|  | Percent of Medicaid covered deliveries | 0.76 (0.50-1.16) | 0.20 |
|  | Percent of county population in rural areas | 1.00 (1.00-1.00) | 0.98 |
|  | Level of neonatal care |  |  |
|  | High | *Reference* |  |
|  | Low | 1.05 (0.89-1.24) | 0.58 |

The fully adjusted model accounted for the random effect of the hospital, the fixed effect of year, and for all covariates listed in the table. The references for the maternal comorbidities are women without those individual conditions. CI, confidence interval.

# eFigure 1: Comparison of complication rates (including and excluding neonatal transfers from the metric numerator) by comorbidity and by level of neonatal care


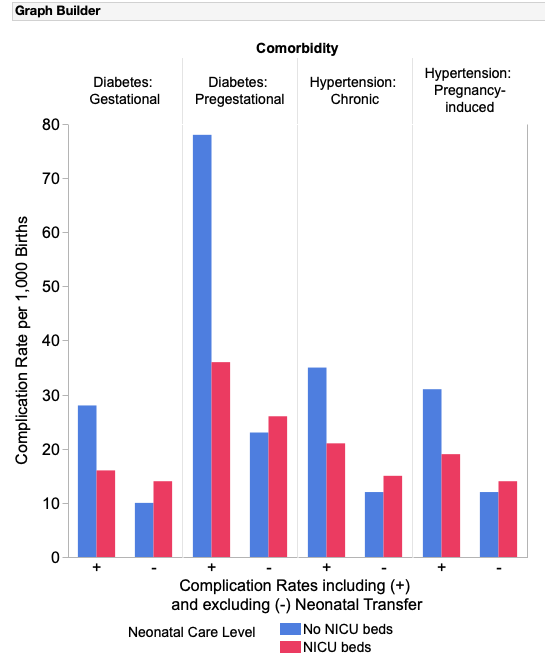


NS

NS

The unexpected newborn complication rates are shown by four comorbidities (gestational diabetes, pregestational diabetes, chronic hypertension, and pregnancy-induced hypertension). Among each comorbidity, the relationship between neonatal transfer and neonatal level of care is demonstrated. P-values for all comparisons are <0.001, expect where noted on the figure. “NS,” non-significant. NICU, neonatal intensive care unit.

# eTable 3: Adjusted odds of severe unexpected newborn complication in patient-level analysis in the model including a state-level fixed effect

|  | Characteristic | Adjusted Odds Ratio (95% CI) | p-value |
| --- | --- | --- | --- |
| Maternal  Characteristics | Maternal age (years) |  |  |
|  | <18 | 0.94 (0.85-1.05) | 0.27 |
|  | 18-24 | *Reference* |  |
|  | 25-29 | 1.02 (0.98-1.05) | 0.3 |
|  | 30-34 | 1.03 (0.99-1.07) | 0.16 |
|  | 35-39 | 1.06 (1.01-1.11) | 0.03 |
|  | 40+ | 1.21 (1.10-1.32) | <0.01 |
|  | Maternal race |  |  |
|  | White | *Reference* |  |
|  | Black | 0.98 (0.94-1.02) | 0.37 |
|  | American Indian/Alaskan Native | 0.93 (0.83-1.03) | 0.17 |
|  | Asian or Pacific Islander | 0.84 (0.77-0.91) | <0.01 |
|  | Ethnicity |  |  |
|  | Hispanic | 0.76 (0.73-0.80) | <0.01 |
|  | Maternal education |  |  |
|  | Less than high school | 1.02 (0.97-1.06) | 0.44 |
|  | High school | *Reference* |  |
|  | Any post-secondary | 0.93 (0.90-0.96) | <0.01 |
|  | Payer at time of delivery |  |  |
|  | Private | *Reference* |  |
|  | Medicaid | 1.17 (1.13-1.21) | <0.01 |
|  | Self-pay | 1.26 (1.16-1.36) | <0.01 |
|  | Other | 1.12 (1.04-1.20) | <0.01 |
|  | Maternal comorbidities |  |  |
|  | Diabetes |  |  |
|  | Pre-gestational | 2.97 (2.73-3.24) | <0.01 |
|  | Gestational | 1.36 (1.29-1.43) | <0.01 |
|  | Hypertension |  |  |
|  | Chronic | 1.47 (1.35-1.59) | <0.01 |
|  | Pregnancy-related | 1.51 (1.44-1.59) | <0.01 |
|  | Tobacco use | 1.31 (1.26-1.36) | <0.01 |
|  | Parity |  |  |
|  | Nulliparous | *Reference* |  |
|  | Multiparous | 0.70 (0.68-0.72) | <0.01 |
| Delivery Characteristics | Gestational age at delivery (weeks) | 0.94 (0.93-0.95) | <0.01 |
|  | Delivery mode |  |  |
|  | Vaginal | *Reference* |  |
|  | Cesarean | 2.11 (2.05-2.16) | <0.01 |
|  | Induction of labor | 0.90 (0.87-0.93) | <0.01 |
|  | Birth weight (grams) | 1.00 (1.00-1.00) | 0.08 |
| Hospital Characteristics | County delivery volume in sample | 1.00 (1.00-1.00) | <0.01 |
|  | Percent of Medicaid covered deliveries | 1.39 (0.99-1.95) | 0.06 |
|  | Percent of county population in rural areas | 1.00 (1.00-1.00) | 0.2 |
|  | Level of neonatal care |  |  |
|  | High | *Reference* |  |
|  | Low | 1.45 (1.29-1.62) | <0.01 |

The adjusted model accounted for the random effect of the hospital, the fixed effects of the state and year, and for all covariates listed in the table. The references for the maternal comorbidities are women without those individual conditions. CI, confidence interval.

# eTable 4: Adjusted odds of severe unexpected newborn complication in patient-level analysis among non-tobacco users

|  | Characteristic | Adjusted Odds Ratio (95% CI) | p-value |
| --- | --- | --- | --- |
| Maternal  Characteristics | Maternal age (years) |  |  |
|  | <18 | 0.94 (0.84-1.04) | 0.25 |
|  | 18-24 | *Reference* |  |
|  | 25-29 | 0.97 (0.93-1.01) | 0.11 |
|  | 30-34 | 0.97 (0.92-1.01) | 0.12 |
|  | 35-39 | 0.98 (0.93-1.04) | 0.58 |
|  | 40+ | 1.14 (1.04-1.26) | 0.01 |
|  | Maternal race |  |  |
|  | White | *Reference* |  |
|  | Black | 0.98 (0.93-1.03) | 0.40 |
|  | American Indian/Alaskan Native | 0.86 (0.76-0.98) | 0.03 |
|  | Asian or Pacific Islander | 0.86 (0.79-0.93) | <0.01 |
|  | Ethnicity |  |  |
|  | Hispanic | 0.76 (0.72-0.80) | <0.01 |
|  | Maternal education |  |  |
|  | Less than high school | 1.02 (0.97-1.07) | 0.51 |
|  | High school | *Reference* |  |
|  | Any post-secondary | 0.92 (0.88-0.95) | <0.01 |
|  | Payer at time of delivery |  |  |
|  | Private | *Reference* |  |
|  | Medicaid | 1.13 (1.09-1.17) | <0.01 |
|  | Self-pay | 1.18 (1.08-1.29) | <0.01 |
|  | Other | 1.08 (1.00-1.17) | 0.05 |
|  | Maternal comorbidities |  |  |
|  | Diabetes |  |  |
|  | Pre-gestational | 2.93 (2.67-3.22) | <0.01 |
|  | Gestational | 1.38 (1.31-1.46) | <0.01 |
|  | Hypertension | *Reference* |  |
|  | Chronic | 1.45 (1.32-1.58) | <0.01 |
|  | Pregnancy-related | 1.52 (1.44-1.60) | <0.01 |
|  | Parity |  |  |
|  | Nulliparous | *Reference* |  |
|  | Multiparous | 0.70 (0.67-0.72) | <0.01 |
| Delivery Characteristics | Gestational age at delivery (weeks) | 0.93 (0.92-0.94) | <0.01 |
|  | Delivery mode |  |  |
|  | Vaginal | *Reference* |  |
|  | Cesarean | 2.19 (2.12-2.25) | <0.01 |
|  | Induction of labor | 0.93 (0.90-0.96) | <0.01 |
|  | Birth weight (grams) | 1.00 (1.00-1.00) | <0.01 |
| Hospital Characteristics | County delivery volume in sample | 1.00 (1.00-1.00) | <0.01 |
|  | Percent of Medicaid covered deliveries | 0.94 (0.70-1.26) | 0.68 |
|  | Percent of county population in rural areas | 1.00 (1.00-1.01) | 0.02 |
|  | Level of neonatal care |  |  |
|  | High | *Reference* |  |
|  | Low | 1.52 (1.35-1.71) | <0.01 |

The adjusted model accounted for the random effect of the hospital, the fixed effect of year, and for all covariates listed in the table. The references for the maternal comorbidities are women without those individual conditions. CI, confidence interval.

# eFigure 2: Distribution of hospital rates of severe unexpected newborn complications among all women and among non-tobacco users


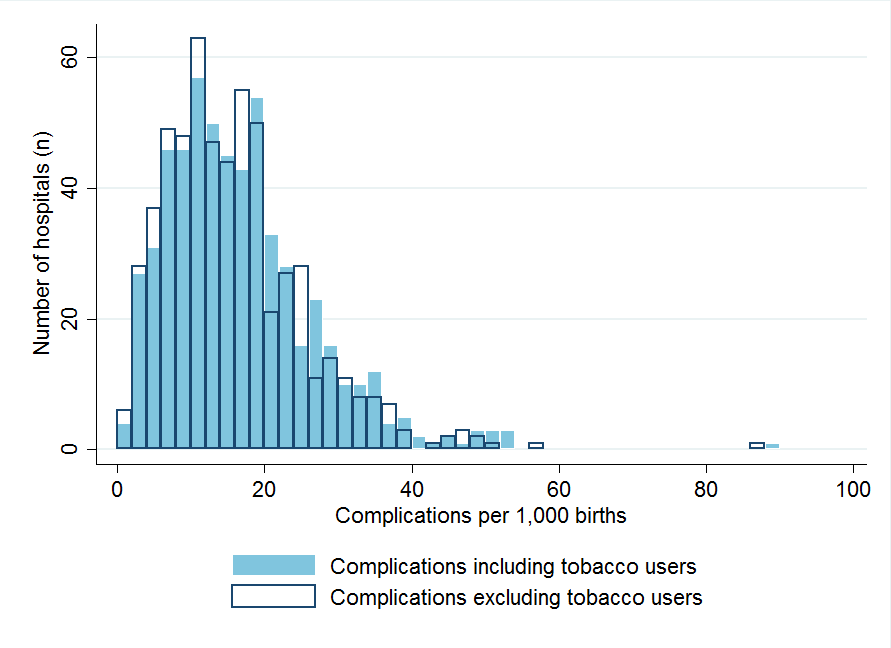


The light blue shaded bars represent the distribution of hospital rates of severe unexpected newborn complications, which includes infants born to tobacco users. The white bars with the dark outline show the distribution of hospital rates of severe unexpected newborn complications shifts when infants born to tobacco are excluded as means of also excluding the majority of illicit drug users.

# eTable 5: Adjusted odds of severe unexpected newborn complication in patient-level analysis excluding maternal transfers

|  | Characteristic | Adjusted Odds Ratio (95% CI) | p-value |
| --- | --- | --- | --- |
| Maternal  Characteristics | Maternal age (years) |  |  |
|  | <18 | 0.94 (0.85-1.04) | 0.23 |
|  | 18-24 | *Reference* |  |
|  | 25-29 | 1.02 (0.98-1.06) | 0.27 |
|  | 30-34 | 1.03 (0.99-1.07) | 0.12 |
|  | 35-39 | 1.07 (1.01-1.12) | 0.02 |
|  | 40+ | 1.20 (1.09-1.32) | <0.01 |
|  | Maternal race |  |  |
|  | White | *Reference* |  |
|  | Black | 0.97 (0.93-1.02) | 0.25 |
|  | American Indian/Alaskan Native | 0.92 (0.83-1.03) | 0.17 |
|  | Asian or Pacific Islander | 0.84 (0.77-0.92) | <0.01 |
|  | Ethnicity |  |  |
|  | Hispanic | 0.77 (0.73-0.80) | <0.01 |
|  | Maternal education |  |  |
|  | Less than high school | 1.02 (0.97-1.06) | 0.46 |
|  | High school | *Reference* |  |
|  | Any post-secondary | 0.93 (0.90-0.96) | <0.01 |
|  | Payer at time of delivery |  |  |
|  | Private | *Reference* |  |
|  | Medicaid | 1.17 (1.13-1.21) | <0.01 |
|  | Self-pay | 1.24 (1.14-1.34) | <0.01 |
|  | Other | 1.11 (1.03-1.19) | 0.01 |
|  | Maternal comorbidities |  |  |
|  | Diabetes |  |  |
|  | Pre-gestational | 2.98 (2.73-3.25) | <0.01 |
|  | Gestational | 1.36 (1.29-1.43) | <0.01 |
|  | Hypertension |  |  |
|  | Chronic | 1.47 (1.35-1.59) | <0.01 |
|  | Pregnancy-related | 1.52 (1.45-1.59) | <0.01 |
|  | Tobacco use | 1.31 (1.26-1.36) | <0.01 |
|  | Parity |  |  |
|  | Nulliparous | *Reference* |  |
|  | Multiparous | 0.70 (0.68-0.72) | <0.01 |
| Delivery Characteristics | Gestational age at delivery (weeks) | 0.94 (0.93-0.95) | <0.01 |
|  | Delivery mode |  |  |
|  | Vaginal | *Reference* |  |
|  | Cesarean | 2.10 (2.05-2.16) | <0.01 |
|  | Induction of labor | 0.90 (0.87-0.93) | <0.01 |
|  | Birth weight (grams) | 0.94 (0.93-0.95) | <0.01 |
| Hospital Characteristics | County delivery volume in sample | 1.00 (1.00-1.00) | <0.01 |
|  | Percent of Medicaid covered deliveries | 0.92 (0.69-1.23) | 0.56 |
|  | Percent of county population in rural areas | 1.00 (1.00-1.01) | 0.03 |
|  | Level of neonatal care |  |  |
|  | High | *Reference* |  |
|  | Low | 1.55 (1.38-1.74) | <0.01 |

The adjusted model accounted for the random effect of the hospital, the fixed effect of year, and for all covariates listed in the table. The references for the maternal comorbidities are women without those individual conditions. CI, confidence interval.

# eFigure 3: Distribution of hospital rates of severe unexpected newborn complications including and excluding maternal transfers from the metric denominator


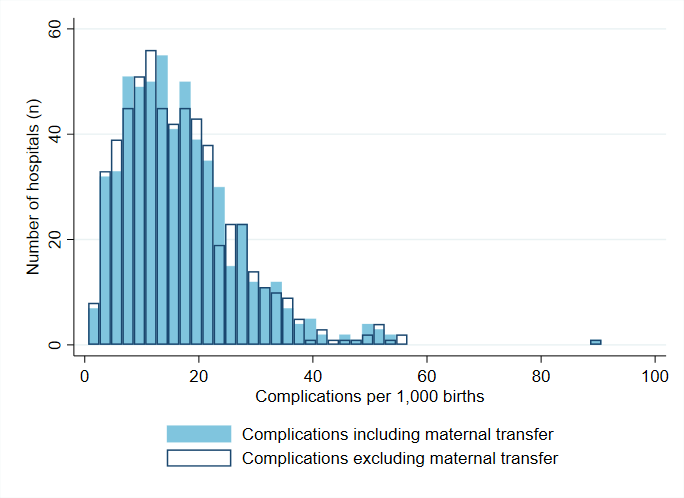


The light blue shaded bars represent the distribution of hospital rates of severe unexpected newborn complications, which includes maternal transfers. The white bars with the dark outline show the distribution of hospital rates of severe unexpected newborn complications shifts when maternal transfers are excluded.

# eTable 6: Comparison on maternal, delivery, and hospitals characteristics between counties with one versus more than one obstetric hospital

| Characteristic | | Counties with 1 hospital with obstetric beds (included in the analysis)  n=1,754,852  N= 563 | Counties with >1 hospital with obstetric beds (excluded in the analysis) n=7,163,084  N= 389 |
| --- | --- | --- | --- |
| Maternal | Maternal age (years) |  |  |
|  | <18 | 29,489 (1.7%) | 94,333 (1.3%) |
|  | 18-24 | 539,193 (30.7%) | 1,627,989 (22.7%) |
|  | 25-29 | 551,920 (31.5%) | 2,073,119 (28.9%) |
|  | 30-34 | 422,216 (24.1%) | 2,107,597 (29.4%) |
|  | 35-39 | 177,659 (10.1%) | 1,036,522 (14.5%) |
|  | 40+ | 34,375 (2.0%) | 223,524 (3.1%) |
|  | Maternal race |  |  |
|  | White | 1,425,367 (81.2%) | 5,312,591 (74.2%) |
|  | Black | 246,396 (14.0%) | 1,145,839 (16.0%) |
|  | American Indian/Alaskan Native | 25,314 (1.4%) | 63,724 (0.9%) |
|  | Asian or Pacific Islander | 57,775 (3.3%) | 640,930 (8.9%) |
|  | Ethnicity |  |  |
|  | Hispanic | 246,262 (14.0%) | 1,924,916 (26.9%) |
|  | Missing | 6,485 (0.4%) | 68,288 (1.0%) |
|  | Maternal education |  |  |
|  | Less than high school | 236,509 (13.5%) | 928,917 (13.0%) |
|  | High school | 504,823 (28.8%) | 1,667,382 (23.3%) |
|  | Any post-secondary | 1,002,600 (57.1%) | 4,462,590 (62.3%) |
|  | Missing | 10,920 (0.6%) | 104,195 (1.5%) |
|  | Payer at time of delivery |  |  |
|  | Medicaid | 794,381 (45.3%) | 2,924,195 (40.8%) |
|  | Private | 812,711 (46.3%) | 3,682,735 (51.4%) |
|  | Self-pay | 60,466 (3.4%) | 24,2377 (3.4%) |
|  | Other | 77,170 (4.4%) | 273,265 (3.8%) |
|  | Missing | 10,124 (0.6%) | 40,512 (0.6%) |
|  | Maternal comorbidities |  |  |
|  | Diabetes |  |  |
|  | Pre-gestational | 12,480 (0.7%) | 51,372 (0.7%) |
|  | Gestational | 94,645 (5.4%) | 422,062 (5.9%) |
|  | Hypertension |  |  |
|  | Chronic | 27,639 (1.6%) | 100,321 (1.4%) |
|  | Pregnancy-induced | 91,963 (5.2%) | 353,537 (4.9%) |
|  | Tobacco use | 203,069 (11.6%) | 358,482 (5.0%) |
|  | Missing | 1,668 (0.1%) | 3,987 (0.1%) |
|  | Parity |  |  |
|  | Nulliparous | 548,780 (31.3%) | 2299,470 (32.1%) |
|  | Multiparous | 1,199,352 (68.3%) | 4826,789 (67.4%) |
|  | Missing | 6,720 (0.4%) | 36,825 (0.5%) |
| Delivery | Gestational age (wks), mean (sd) | 39.3 (1.5) | 39.3 (1.4) |
|  | Delivery mode |  |  |
|  | Vaginal | 1,254,846 (71.5%) | 5,025,638 (70.2%) |
|  | Cesarean | 499,182 (28.4%) | 2,135,563 (29.8%) |
|  | Missing | 824 (<0.1%) | 1,883 (<0.1%) |
|  | Induction of labor | 519,719 (29.6%) | 1,800,440 (25.1%) |
|  | Missing | 679 (<0.1%) | 2,672 (<0.1%) |
|  | Infant birth weight (grams), mean (sd) | 3,412 (436) | 3,406 (431) |
|  | Missing | 256 (<0.1%) | 3,142 (<0.1%) |
|  | Maternal transfer | 3,101 (0.2%) | 12,592 (0.2%) |
|  | Missing | 684 (<0.1%) | 2,264 (<0.1%) |
| County | Average annual hospital delivery volume | 2,043 (1,353, 3,508) | 3,208 (1,527, 6,740) |
|  | Percent of births covered by Medicaid | 49.0 (37.2, 60.5) | 40.8 (32.0, 51.2) |
|  | Percent of county population in rural area | 37.2 (24.2, 53.9) | 13.5 (4.9, 27.0) |
|  | Hospitals with NICU beds | 209 (37.1%) | 307 (78.9%) |

All maternal and delivery characteristics are presented as number of deliveries (n (% of deliveries)), unless otherwise noted. County characteristics are presented as median (interquartile range) or n (%).

NICU, neonatal intensive care unit; sd, standard deviation.

P-values <0.001 for all comparisons. NICU, neonatal intensive care unit.

# eTable 7: Comparison of neonatal complications between counties with one versus more than one obstetric hospital

|  | Deliveries in counties with 1 hospital with obstetric beds (included in the analysis)  n=1,754,852 | Deliveries in counties with >1 hospital with obstetric beds (excluded in the analysis) n=7,163,084 | p-value |
| --- | --- | --- | --- |
| Neonatal complications | 24,604 (1.4%) | 719,21 (1.0%) | <0.001 |
| Transfer | 14,678 (0.8%) | 32,201 (0.5%) | <0.001 |
| Assisted ventilation ≥6 hours | 8,259 (0.5%) | 29,058 (0.4%) | <0.001 |
| Seizure | 627 (<0.1%) | 1,563 (<0.1%) | <0.001 |
| Neonatal death | 1,035 (0.1%) | 6,835 (0.1%) | <0.001 |
| 5-minute Apgar ≤3 | 4,365 (0.2%) | 16,217 (0.2%) | <0.001 |
| Missing | 3,960 (0.2%) | 10,480 (0.1%) | <0.001 |

All complication data are presented as n (% of deliveries).

# eFigure 4: Comparison of distributions of complication rates including and excluding transfer from the metric numerator among counties with more than one obstetric hospital


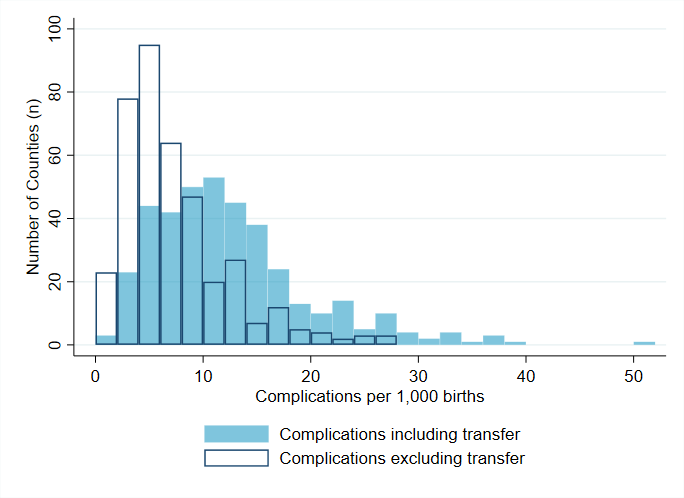


The light blue shaded bars represent the distribution of county rates of severe unexpected newborn complications, which includes neonatal transfer as a complication per the Joint Commission metric. The median and interquartile range (IQR) of the complication rate was 11.3 (7.1-15.7). The white bars with the dark blue outline show how the distribution of county rates of severe unexpected newborn complications shifts when neonatal transfers are excluded. The median and IQR excluding transfers was reduced to 6.0 (3.9-9.4).

# eTable 8: Between-county variation estimations in counties with more than one obstetric hospital

| Model | Intraclass Correlation Coefficient (95% CI) |
| --- | --- |
| Year only | 9.9% (8.7-11.3%) |
| Year, patient factors | 9.4% (8.2-10.7%) |
| Year, patient, county factors | 8.3% (7.2-9.5%) |

CI, confidence interval.

# eTable 9: Adjusted odds of severe unexpected newborn in the patient-level analysis in counties with more than one obstetric hospital

|  | Characteristic | Adjusted Odds Ratio (95% CI) | p-value |
| --- | --- | --- | --- |
| Maternal  Characteristics | Maternal age (years) |  |  |
|  | <18 | 1.13 (1.06-1.20) | 0.00 |
|  | 18-24 | *Reference* |  |
|  | 25-29 | 0.98 (0.96-1.00) | 0.09 |
|  | 30-34 | 0.95 (0.92-0.97) | 0.00 |
|  | 35-39 | 0.97 (0.95-1.00) | 0.06 |
|  | 40+ | 1.06 (1.01-1.11) | 0.02 |
|  | Maternal race |  |  |
|  | White | *Reference* |  |
|  | Black | 1.06 (1.04-1.09) | 0.00 |
|  | American Indian/Alaskan Native | 1.05 (0.98-1.13) | 0.19 |
|  | Asian or Pacific Islander | 0.83 (0.80-0.86) | 0.00 |
|  | Ethnicity |  |  |
|  | Hispanic | 0.81 (0.79-0.83) | 0.00 |
|  | Maternal education |  |  |
|  | Less than high school | 1.03 (1.00-1.06) | 0.02 |
|  | High school |  |  |
|  | Any post-secondary | 0.93 (0.91-0.95) | 0.00 |
|  | Payer at time of delivery |  |  |
|  | Private | *Reference* |  |
|  | Medicaid | 1.21 (1.18-1.23) | 0.00 |
|  | Self-pay | 1.28 (1.22-1.33) | 0.00 |
|  | Other | 1.44 (1.39-1.50) | 0.00 |
|  | Maternal comorbidities |  |  |
|  | Diabetes |  |  |
|  | Pre-gestational | 2.70 (2.57-2.84) | 0.00 |
|  | Gestational | 1.36 (1.32-1.40) | 0.00 |
|  | Hypertension |  |  |
|  | Chronic | 1.53 (1.46-1.61) | 0.00 |
|  | Pregnancy-related | 1.50 (1.45-1.54) | 0.00 |
|  | Tobacco use | 1.38 (1.34-1.42) | 0.00 |
|  | Parity |  |  |
|  | Nulliparous |  |  |
|  | Multiparous | 0.67 (0.66-0.68) | 0.00 |
| Delivery Characteristics | Gestational age at delivery (weeks) | 0.96 (0.95-0.96) | 0.00 |
|  | Delivery mode |  |  |
|  | Vaginal | *Reference* |  |
|  | Cesarean | 2.19 (2.16-2.22) | 0.00 |
|  | Induction of labor | 1.05 (1.03-1.06) | 0.00 |
|  | Infant birth weight (grams) | 1.00 (1.00-1.00) | 0.00 |
| Hospital Characteristics | Delivery volume | 1.00 (1.00-1.00) | 0.01 |
|  | Percent of Medicaid covered deliveries | 0.67 (0.44-1.01) | 0.06 |
|  | Percent of county population in rural areas | 1.01 (1.00-1.01) | 0.02 |
|  | Level of neonatal care |  |  |
|  | High | *Reference* |  |
|  | Low | 1.30 (1.09-1.54) | 0.00 |

The adjusted model accounted for the random effect of the county, the fixed effect of year, and for all covariates listed in the table. The references for the maternal comorbidities are women without those individual conditions. CI, confidence interval.
